# NRP1 promotes hepatic fibrosis by regulating Src/Rab7-mediated lysosomal degradation of TβRI

**DOI:** 10.1038/s41420-026-03220-w

**Published:** 2026-06-28

**Authors:** Zhe Jia, Xiaodong Song, Jie Bai

**Affiliations:** 1 Department of General Surgery, Beijing Friendship Hospital, Capital Medical University & State Key Lab of Digestive Health & National Clinical Research Center for Digestive Diseases, Beijing, China; 2 Department of Neurology, The First Affiliated Hospital of Chongqing Medical University, Chongqing, China; 3 Department of Infectious Diseases, The First Affiliated Hospital of Chongqing Medical University, Chongqing, China

**Keywords:** Liver fibrosis, Growth factor signalling

## Abstract

**Background and Aims:**

Activation of hepatic stellate cells (HSCs) and subsequent dysregulation of the TGF-β1 signaling pathway are central to the progression of hepatic fibrosis. Neuropilin-1 (NRP1) has been identified as a co-receptor involved in multiple signaling pathways, yet its role in modulating TGF-β receptor stability in HSCs remains to be fully elucidated. This study investigated the regulatory role of the NRP1/Src/Rab7 axis in TβRI lysosomal degradation during HSC activation.

**Methods:**

Hepatic fibrosis was induced in male C57BL/6 mice via intraperitoneal CCl_4_ injection for four weeks. HSC-specific knockdown of *Nrp1* was achieved using an AAV8-pLrat-sh*Nrp1* vector. TβRI stability and lysosomal degradation were evaluated through cycloheximide (CHX) chase assays and chloroquine treatment. The impact on the TGF-β1/Smad signaling pathway was assessed both in vitro and in vivo.

**Results:**

*Nrp1* silencing in primary HSCs reduced TβRI protein levels and attenuated p-Smad2/3 signaling without affecting mRNA levels. Consistently, NRP1 deficiency accelerated lysosomal degradation of TβRI, accompanied by increased TβRI–LAMP1 colocalization and enhanced trafficking of TβRI into Rab7-positive late endosomes. Mechanistically, NRP1 loss diminished Src kinase activity and consequently reduced Src-dependent tyrosine phosphorylation of Rab7, leading to elevated Rab7-GTP levels and enhanced late endosomal sorting of TβRI. *Rab7* knockdown or Src reactivation partially rescued TβRI stability and restored downstream signaling. In vivo, HSC-specific *Nrp1* knockdown attenuated CCl_4_-induced fibrosis and reduced TβRI levels, while Rab7 inhibition partially reversed these effects.

**Conclusions:**

These data suggest that the NRP1/Src/Rab7 axis contributes to TβRI stability by modulating its lysosomal degradation pathway. Targeting this axis may provide a potential approach for intervening in the progression of hepatic fibrosis.

**Figure:**
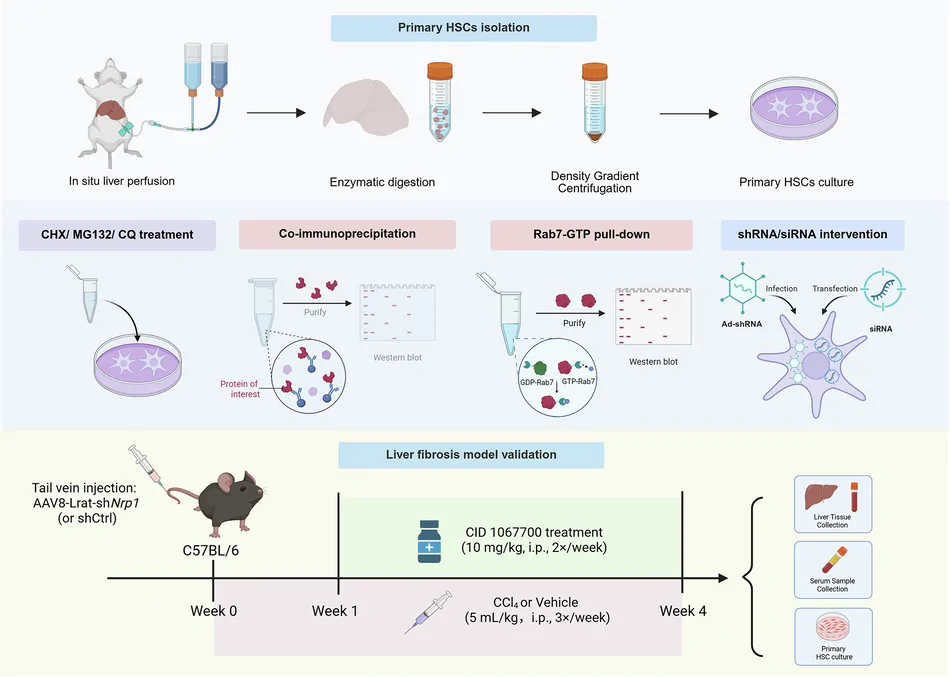

## Introduction

Liver fibrosis is a pathological consequence of chronic liver injury, characterized by the excessive accumulation of extracellular matrix (ECM) [1]. The activation of hepatic stellate cells (HSCs) and the subsequent deposition of collagen are central events in this process, eventually leading to organ failure [2–4]. Currently, effective medical therapies for liver fibrosis remain limited, partly because the underlying pathogenic mechanisms are not fully understood [4, 5]. Therefore, it is important to explore the molecular mechanisms of fibrosis to develop new therapeutic strategies.

Neuropilin-1 (NRP1) is a single-pass transmembrane non-tyrosine kinase co-receptor, originally identified for its roles in axonal guidance and angiogenesis [6]. Recent studies have expanded its function, showing that NRP1 can bind to various growth factors (such as VEGF, PDGF, and TGF-β) and their respective receptors to regulate cell proliferation, migration, and tissue fibrosis [7, 8]. Our previous studies established that NRP1 expression is markedly upregulated in activated HSCs, where it serves as a critical driver of liver fibrosis [9]. We also demonstrated that the deubiquitinase USP9X maintains NRP1 protein levels by preventing its proteasomal degradation [9]. While NRP1 is known to be a vital modulator of TGF-β signaling, the precise mechanisms by which it coordinates intracellular signaling events and whether it functions by directing the endosomal transport of receptors remain to be elucidated [10].

TGF-β is a master cytokine in the pathogenesis of organ fibrosis, initiating signaling by binding to a complex of type I (TβRI) and type II (TβRII) receptors on the cell membrane [11, 12]. Evidence indicates that the intracellular distribution of these receptors following endocytosis determines the strength and duration of the signal: some receptors are recycled back to the plasma membrane, while others are transported to lysosomes for degradation [13, 14]. Rab family proteins play a central role in regulating these transport processes [15]. As members of the small GTPase family, different Rab proteins are localized to specific endosomal membranes and direct receptor trafficking to distinct organelles [15, 16]. Multiple factors have been shown to modulate the activity of Rab family members, thereby altering the intracellular localization and degradation of receptors [17]. However, whether NRP1 participates in the intracellular transport of TGF-β receptors and whether it functions by influencing Rab-mediated receptor sorting pathways remains to be clarified.

In this study, using primary mouse HSCs and mouse models of liver fibrosis, we investigated the effect of NRP1 on the subcellular distribution of TβRI. We found that NRP1 deficiency led to increased localization of TβRI in lysosomes and its subsequent degradation. Mechanistically, NRP1 promotes Src kinase activation, which in turn mediates the phosphorylation of Rab7, thereby restricting Rab7-mediated trafficking of TβRI to lysosomes. Our findings suggest that modulating NRP1 and its associated receptor transport pathways may provide new therapeutic strategies for the treatment of liver fibrosis.

## Results

### 1 NRP1 maintains TGF-β/Smad signaling activation by regulating TβRI in HSCs

**Fig. 1:**
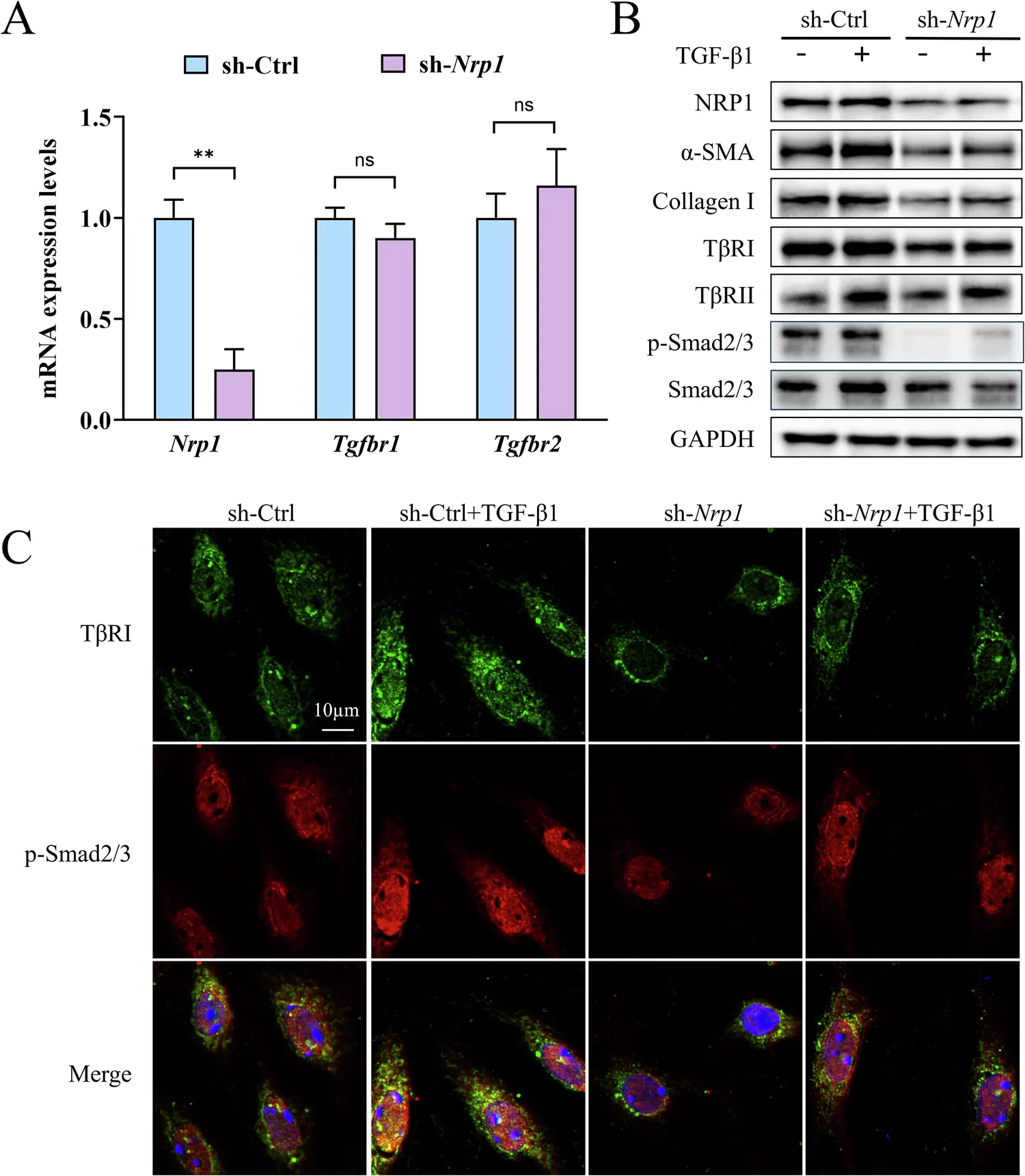
NRP1 maintains TGF-β/Smad signaling activation by regulating TβRI in HSCs. **A** Quantitative PCR (qPCR) analysis of *Nrp1*, *Tgfbr1*, and *Tgfbr2* mRNA expression levels in primary mouse HSCs transfected with control shRNA (sh-Ctrl) or *Nrp1* shRNA (sh-*Nrp1*). **B** Western blot analysis of NRP1, α-SMA, Collagen I, TβRI, TβRII, p-Smad2/3, and total Smad2/3 expression levels in primary mouse HSCs transfected with sh-Ctrl or sh-*Nrp1* in the presence or absence of TGF-β1 stimulation (5 ng/mL) for 48 h. **C** Representative immunofluorescence images showing the expression of TβRI (green) and the nuclear translocation of p-Smad2/3 (red) in HSCs. Scale bars = 10 μm. Data are presented as mean ± SD. Statistical significance was determined by two-tailed Student’s *t* test. ns, not significant; * *p* < 0.05; ** *p* < 0.01.

To assess whether NRP1 influences TGF-β/Smad signaling in hepatic stellate cells, we silenced *Nrp1* using shRNA in cultured primary mouse HSCs. qRT-PCR confirmed efficient knockdown of *Nrp1* mRNA. In contrast, mRNA levels of *Tgfbr1* and *Tgfbr2* (encoding TβRI and TβRII, respectively) remained unchanged (Fig. 1A). These results suggest that NRP1 modulates these receptors through a post-transcriptional mechanism rather than at the transcriptional level.

We next examined key components of the pathway at the protein level. Immunoblotting showed that *Nrp1* knockdown significantly reduced TβRI protein levels, which were accompanied by decreased phosphorylation of Smad2 and Smad3 (p-Smad2/3). Consistently, the expression of fibrogenic markers, including α-SMA and Collagen I, was also reduced (Fig. 1B). Notably, TβRII protein levels remained unaffected (Fig. 1B), indicating a more selective effect on TβRI. Furthermore, exogenous TGF-β1 stimulation failed to fully restore downstream signaling and fibrogenic responses in NRP1-deficient cells (Fig. 1B), suggesting that the signaling defect was primarily due to receptor deficiency. Consistent with these findings, immunofluorescence revealed lower TβRI intensity and reduced nuclear accumulation of p-Smad2/3 upon *Nrp1* knockdown (Fig. 1C). Together, these data indicate that NRP1 is essential for maintaining TβRI protein levels in HSCs, thereby sustaining TGF-β/Smad signaling and the subsequent fibrogenic process.

### 2 NRP1 restrains the lysosomal degradation of TβRI in HSCs

**Fig. 2:**
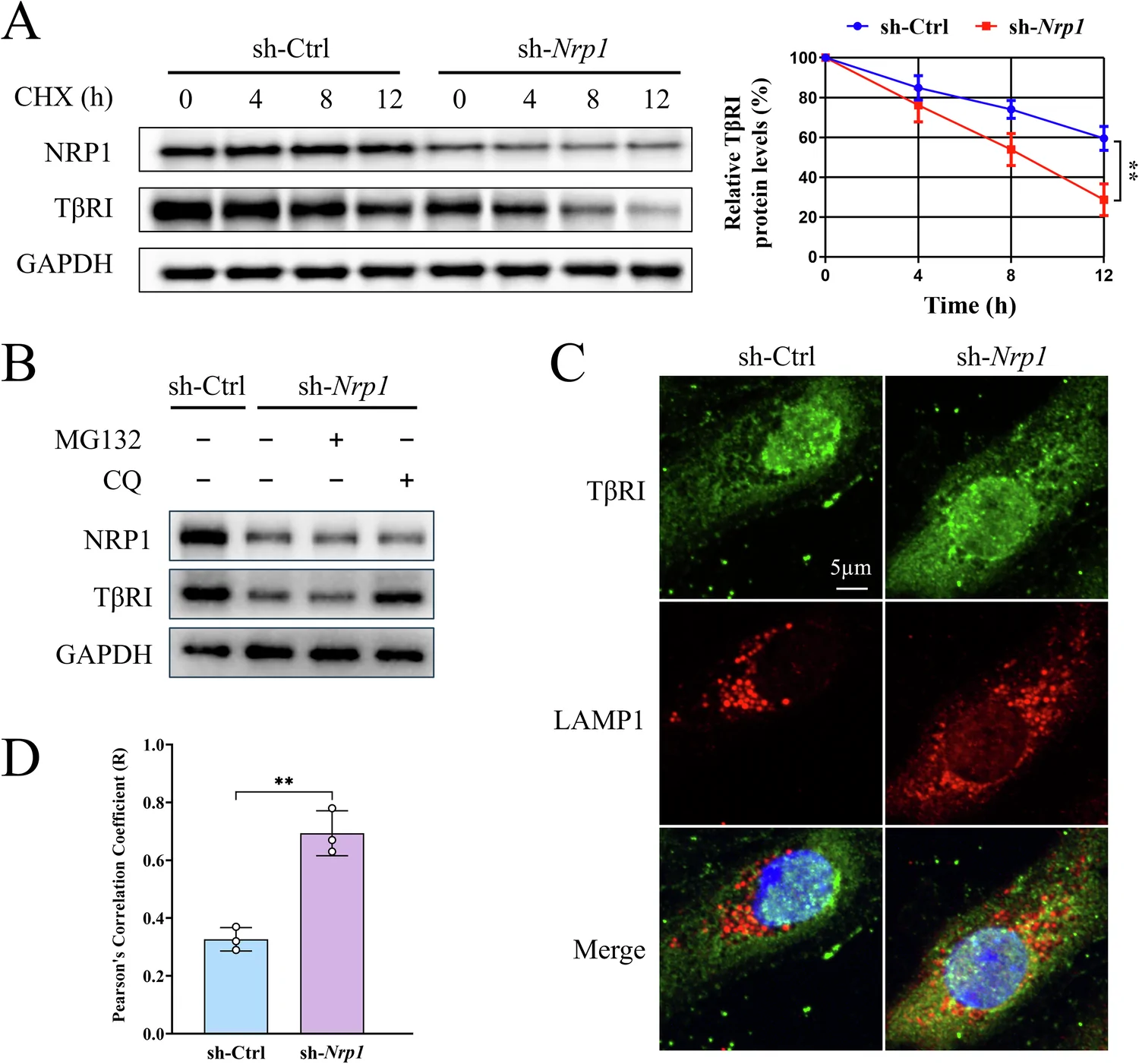
NRP1 restrains the lysosomal degradation of TβRI in HSCs. **A** Half-life analysis and quantification of TβRI in *Nrp1*-knockdown primary mouse HSCs. All cell groups were transfected with control shRNA (sh-Ctrl) or *Nrp1* shRNA (sh-*Nrp1*), and then treated with cycloheximide (CHX, 10 μg/mL) for 0, 4, 8, and 12 h. **B** Western blot analysis of NRP1 and TβRI expression levels in primary mouse HSCs transfected with sh-Ctrl or sh-*Nrp1* and treated with the proteasome inhibitor MG132 (10 μM) or the lysosomal inhibitor chloroquine (CQ, 50 μM) for 6 h. **C** Immunofluorescence staining showing the colocalization of TβRI (green) and the lysosomal marker LAMP1 (red) in primary mouse HSCs transfected with sh-Ctrl or sh-*Nrp1* and treated with CQ (50 μM). **D** Quantitative analysis of colocalization between TβRI and LAMP1 as determined by Pearson’s correlation coefficient (R). Data are presented as mean ± SD. Statistical significance was determined by two-way repeated measures ANOVA followed by Sidak’s multiple comparisons test for post-hoc analysis for (**A**), and by two-tailed Student’s *t* test for (**D**). ns, not significant; * *p* < 0.05; ** *p* < 0.01.

Having confirmed that NRP1 maintains TβRI protein levels without affecting its mRNA expression, we sought to determine the mechanism by which NRP1 regulates TβRI at the post-translational level. We first performed cycloheximide (CHX) chase assays to evaluate TβRI protein stability in primary mouse HSCs. The results demonstrated that *Nrp1* knockdown significantly accelerated the degradation of TβRI, leading to a marked decrease in its half-life (Fig. 2A). To identify the specific degradation pathway involved, we utilized the lysosomal inhibitor chloroquine and the proteasome inhibitor MG132. Notably, chloroquine largely restored TβRI protein levels diminished by NRP1 deficiency, whereas MG132 exerted minimal effects (Fig. 2B). These results indicate that lysosome-mediated degradation is the predominant route for TβRI loss in the absence of NRP1.

Consistent with this, after blocking lysosomal degradation with chloroquine, immunofluorescence co-staining demonstrated a significant increase in the colocalization of TβRI with the lysosomal marker LAMP1 in *Nrp1*-silenced cells (Fig. 2C). This increase was further validated by quantitative colocalization analysis (Fig. 2D). Collectively, these results indicate that NRP1 inhibits the lysosomal trafficking and degradation of TβRI, thereby preserving TβRI protein abundance in HSCs.

### 3 NRP1 prevents the sorting of TβRI into Rab7⁺ late endosomes

**Fig. 3:**
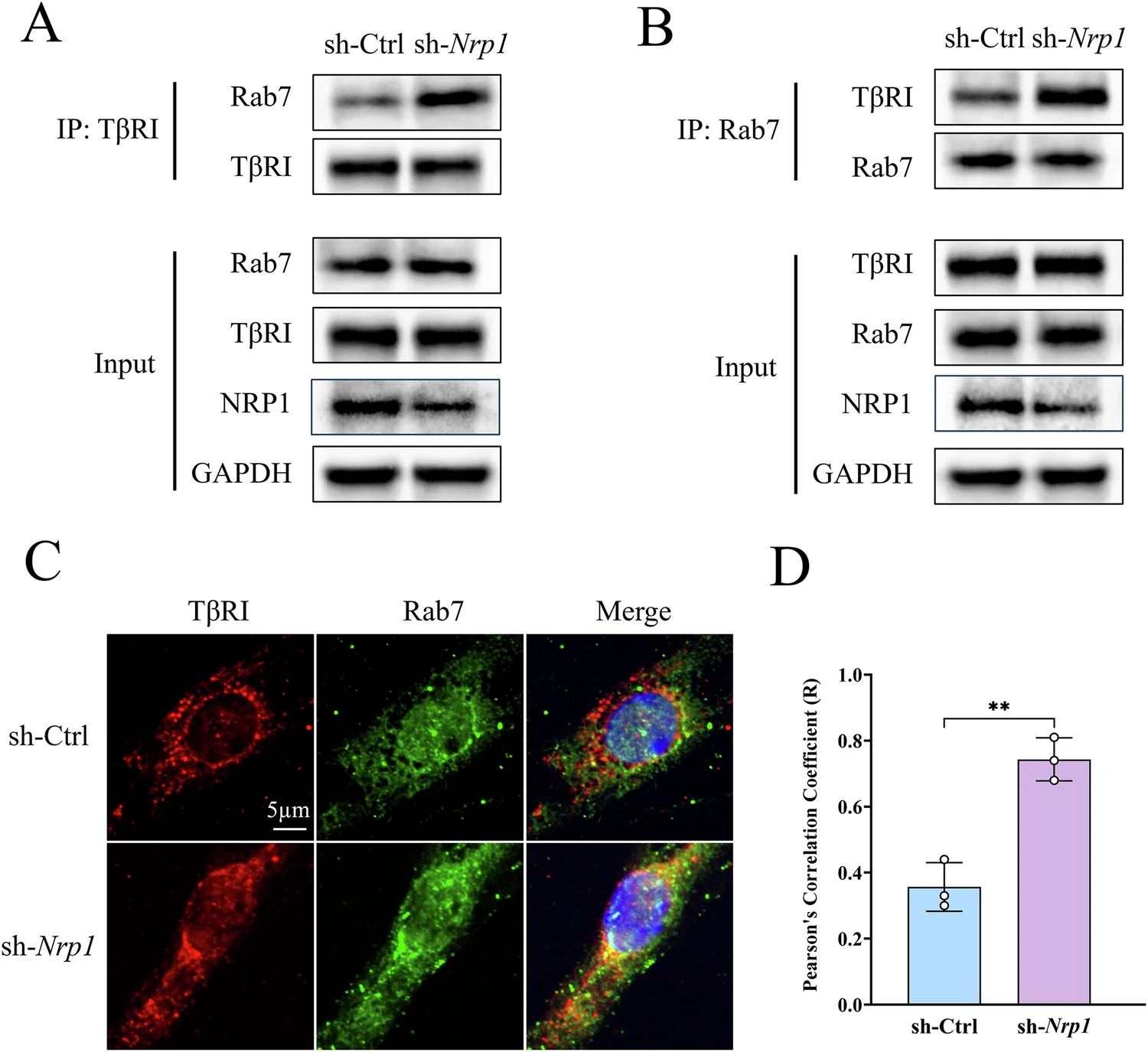
NRP1 prevents the sorting of TβRI into Rab7-positive late endosomes in HSCs. Endogenous co-immunoprecipitation (Co-IP) analysis of the interaction between TβRI and Rab7. Primary mouse HSCs were transfected with control shRNA (sh-Ctrl) or *Nrp1* shRNA (sh-*Nrp1*) and treated with the lysosomal inhibitor chloroquine (CQ, 50 μM) for 6 h. Cell lysates were subjected to immunoprecipitation with an anti-TβRI antibody, followed by immunoblotting with antibodies against Rab7 and TβRI (**A**). Cell lysates were subjected to immunoprecipitation with an anti-Rab7 antibody, followed by immunoblotting with antibodies against TβRI and Rab7 (**B**). **C** Representative immunofluorescence images showing the colocalization of TβRI (green) and the late endosomal marker Rab7 (red) in sh-Ctrl or sh-*Nrp1* HSCs treated with CQ (50 μM). **D** Quantitative analysis of colocalization between TβRI and Rab7 as determined by Pearson’s correlation coefficient (R). Data are presented as the mean ± SD. Statistical significance was determined by two-tailed Student’s *t* test. ns, not significant; * *p* < 0.05; ** *p* < 0.01.

Given our preceding findings that TβRI undergoes lysosomal degradation upon NRP1 loss, we focused on Rab7, a key molecular switch governing late endosomal trafficking. Endogenous Co-IP assays revealed that, under lysosomal inhibition, NRP1 deficiency significantly enhanced the protein interaction between TβRI and Rab7 compared to the control group (Fig. 3A, B). Confocal immunofluorescence analysis further confirmed that TβRI accumulated within Rab7-positive punctate structures in *Nrp1*-silenced HSCs, as evidenced by a significantly higher colocalization coefficient than in control cells (Fig. 3C, D). Collectively, these results demonstrate that NRP1 serves to restrain the sorting of TβRI into the Rab7-positive late endosomal pathway, thereby protecting the receptor from degradation.

### 4 *Rab7* knockdown partially rescues NRP1 deficiency-mediated TβRI degradation and signaling inhibition

**Fig. 4:**
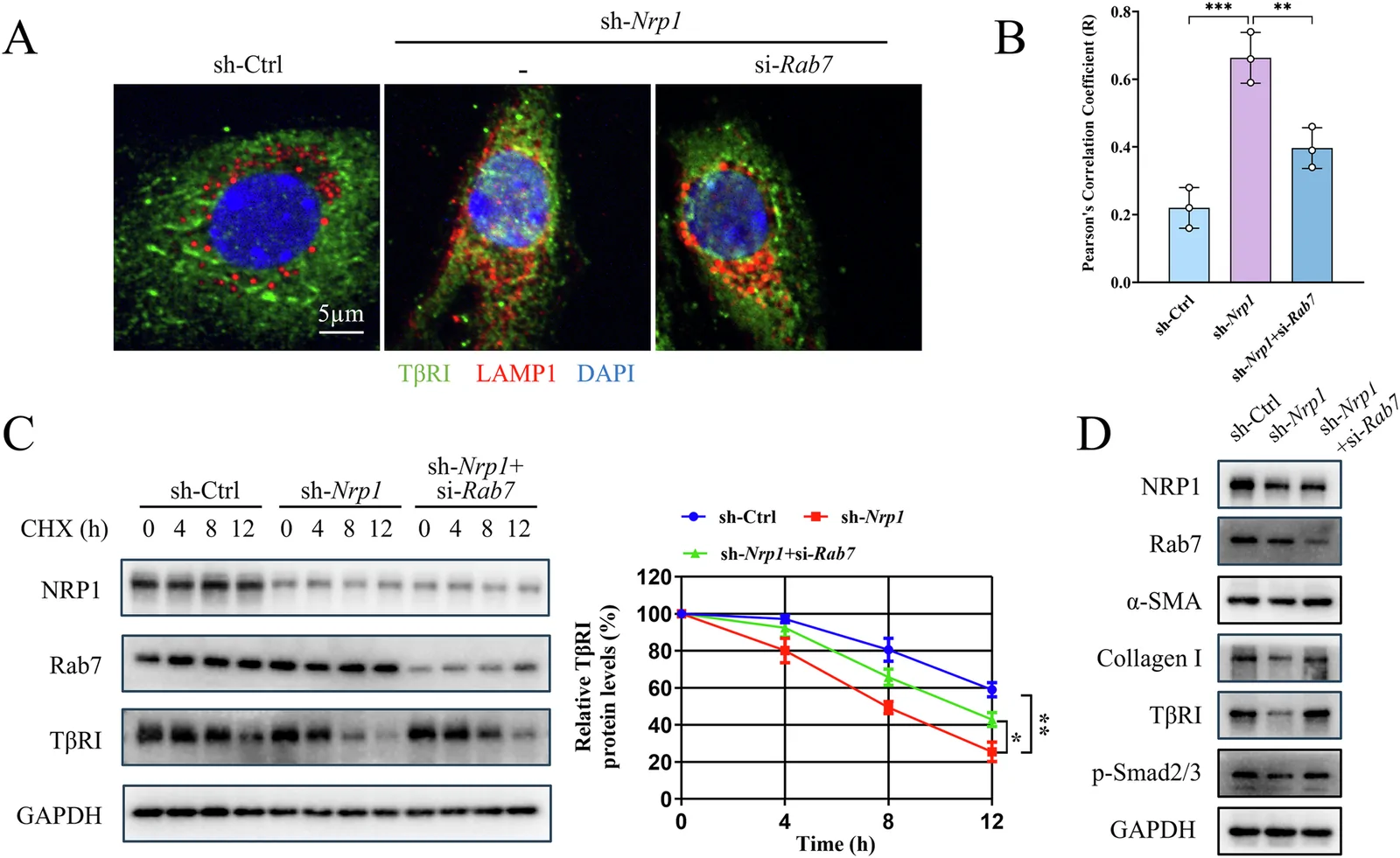
Rab7 knockdown partially rescues NRP1 deficiency-mediated TβRI degradation and signaling inhibition in HSCs. **A** Representative immunofluorescence images showing the colocalization of TβRI (green) and the lysosomal marker LAMP1 (red) in primary mouse HSCs transfected with control shRNA (sh-Ctrl), *Nrp1* shRNA (sh-*Nrp1*), or co-transfected with sh-*Nrp1* and *Rab7* siRNA (si-*Rab7*). All groups were treated with the lysosomal inhibitor chloroquine (50 μM) for 6 h. **B** Quantitative analysis of colocalization between TβRI and LAMP1 as determined by Pearson’s correlation coefficient (R). **C** Half-life analysis and quantification of TβRI in the indicated HSC groups. All cell groups were treated with cycloheximide (CHX, 10 μg/mL) for 0, 4, 8, and 12 h, and subjected to immunoblotting. **D** Western blot analysis of NRP1, Rab7, α-SMA, Collagen I, TβRI, and p-Smad2/3 expression levels in primary mouse HSCs transfected with sh-Ctrl, sh-*Nrp1*, or sh-*Nrp1* plus si-*Rab7*. Data are presented as the mean ± SD of three independent experiments. Statistical significance was determined by one-way ANOVA followed by Tukey’s multiple comparisons test for (**B**), and by two-way repeated measures ANOVA followed by Sidak’s multiple comparisons test for (**C**). ns, not significant; * *p* < 0.05; ** *p* < 0.01; *** *p* < 0.001.

To investigate the functional role of Rab7 in TβRI lysosomal degradation induced by NRP1 loss, we co-silenced *Nrp1* and *Rab7* in primary HSCs. Under lysosomal inhibition with chloroquine, confocal immunofluorescence analysis showed that NRP1 deficiency significantly increased the colocalization of TβRI with the lysosomal marker LAMP1, and this increase was markedly attenuated by *Rab7* knockdown (Fig. 4A, B). Cycloheximide (CHX) chase assays revealed that while *Nrp1* knockdown shortened the protein half-life of TβRI, silencing *Rab7* partially prolonged its stability and slowed its degradation (Fig. 4C). Furthermore, Western blot analysis demonstrated that *Rab7* knockdown partially restored TβRI protein levels and downstream p-Smad2/3 signaling, which were otherwise suppressed by NRP1 loss. This was accompanied by a corresponding recovery in the expression of HSC activation markers, α-SMA and Collagen I (Fig. 4D). Collectively, these results suggest that the accelerated lysosomal degradation of TβRI resulting from NRP1 deficiency is, at least in part, dependent on Rab7-mediated trafficking.

### 5 NRP1 regulates TβRI lysosomal sorting and HSC activation via Src-mediated Rab7 tyrosine phosphorylation

**Fig. 5:**
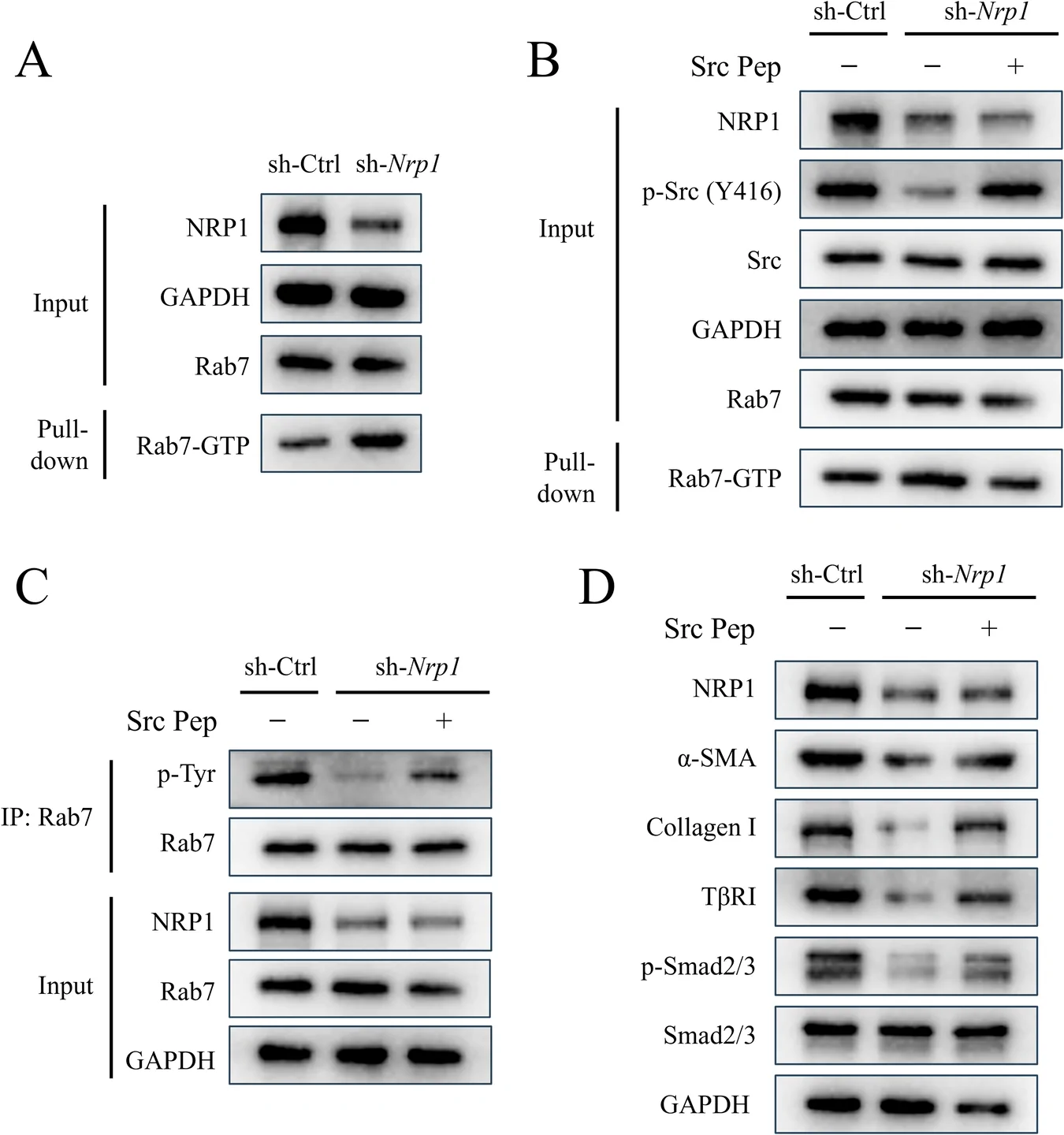
NRP1 regulates TβRI lysosomal sorting and HSC activation via Src-mediated Rab7 tyrosine phosphorylation. **A** Rab7-GTP pull-down assay in primary mouse HSCs transfected with control shRNA (sh-Ctrl) or *Nrp1* shRNA (sh-*Nrp1*). Cell lysates were incubated with GST-RILP-PBD beads to capture active Rab7-GTP, followed by immunoblotting with an anti-Rab7 antibody. Primary mouse HSCs transfected with sh-Ctrl or sh-*Nrp1* were treated with or without a Src-activating peptide (Src Pep: EPQpYEEIPIYL; 10 μM) for 24 h. Western blot analysis of p-Src (Y416), total Src, NRP1, and Rab7 levels; active Rab7-GTP was determined by pull-down assay (**B**). Immunoprecipitation (IP) analysis of Rab7 tyrosine phosphorylation. Cell lysates were subjected to IP with an anti-Rab7 antibody, followed by immunoblotting with an anti-phospho-Tyrosine (p-Tyr) antibody (**C**). Western blot analysis of HSC activation markers (α-SMA, Collagen I) and TGF-β/Smad signaling components (TβRI, p-Smad2/3, total Smad2/3) (**D**).

Rab7 drives late endosome-to-lysosome trafficking primarily by recruiting effector proteins in its GTP-bound state; accordingly, we further investigated whether NRP1 influences Rab7 activation. Rab7-GTP pull-down assays showed that *Nrp1* knockdown significantly increased Rab7-GTP levels while total Rab7 protein remained unchanged (Fig. 5A), indicating that Rab7 is hyperactivated upon NRP1 loss.

Given the reported association between NRP1 and Src family kinases, we examined the activation status of Src. Western blot analysis revealed that *Nrp1* knockdown led to a marked decrease in Src kinase activity, as indicated by diminished p-Src (Tyr416) levels (Fig. 5B). To establish a functional link, we employed a Src-activating peptide (EPQpYEEIPIYL), which effectively restored Src activation in NRP1-deficient cells. Consequently, the hyperactivation of Rab7, as reflected by elevated Rab7-GTP levels, induced by NRP1 loss, was partially reversed by the reactivation of Src (Fig. 5B). These findings suggest that Src signaling acts as a negative regulator of Rab7 activation in this context.

To explore the specific mechanism by which Src modulates Rab7, we assessed Rab7 tyrosine phosphorylation via immunoprecipitation. *Nrp1* knockdown diminished the Rab7 tyrosine phosphorylation signal, which was subsequently restored by the Src-activating peptide (Fig. 5C). These data suggest that NRP1 deficiency suppresses Src activity, which reduces Rab7 phosphorylation and thereby triggers the hyperactivation of Rab7. Furthermore, Src activation partially rescued the TβRI protein loss and p-Smad2/3 signaling inhibition caused by *Nrp1* knockdown, accompanied by a recovery in α-SMA and Collagen I expression (Fig. 5D). Collectively, these findings suggest that NRP1 deficiency inhibits Src-mediated Rab7 tyrosine phosphorylation, leading to elevated Rab7-GTP levels. This promotes TβRI sorting into the lysosomal pathway, ultimately suppressing TGF-β/Smad signaling and inhibiting the HSC activation phenotype.

### 6 HSC-specific NRP1 knockdown reduces TβRI protein levels and alleviates hepatic fibrosis in mice

**Fig. 6:**
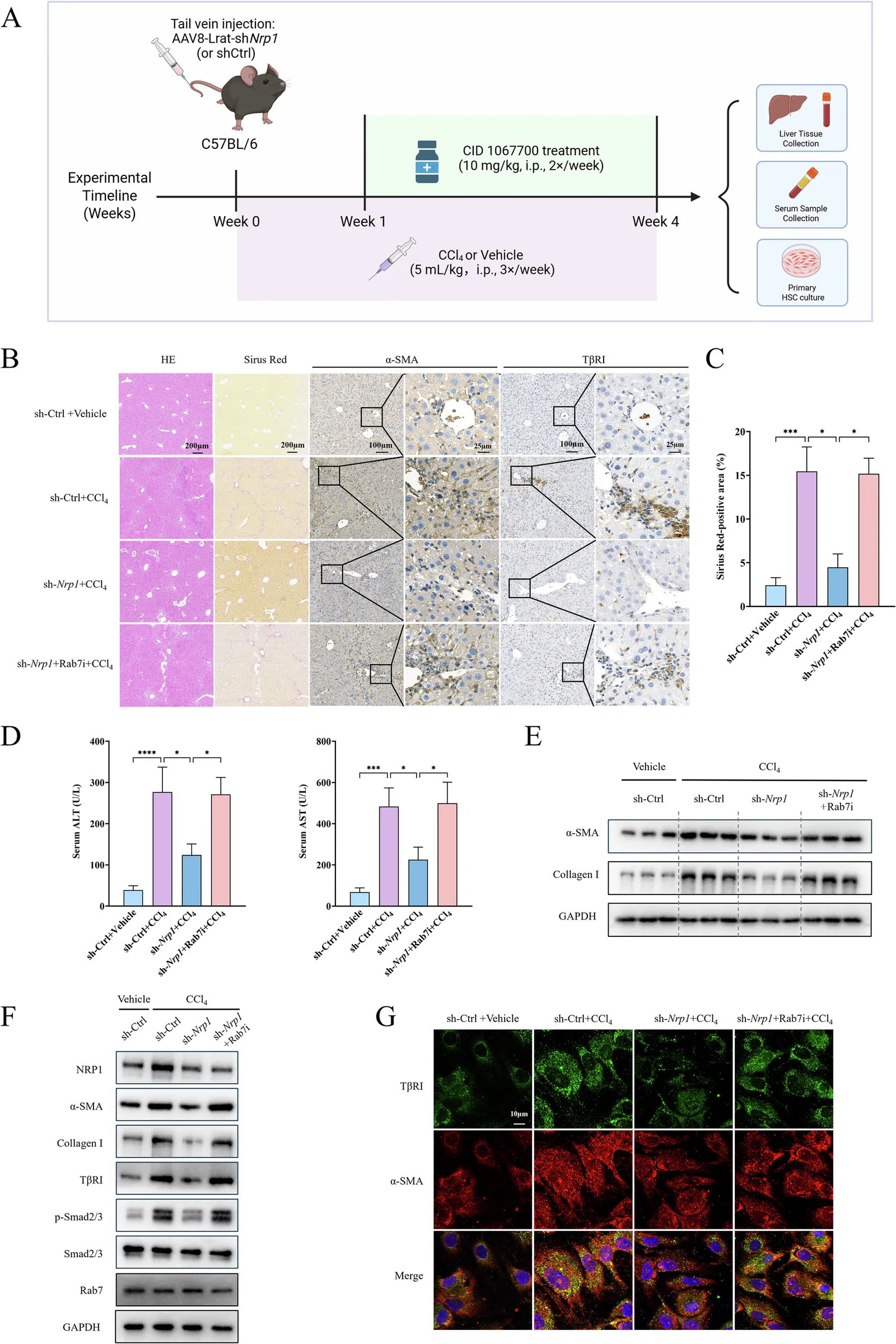
HSC-specific NRP1 knockdown reduces TβRI protein levels and alleviates hepatic fibrosis in mice. **A** Experimental timeline and strategy for HSC-specific *Nrp1* knockdown and pharmacological rescue. C57BL/6 mice received tail vein injections of AAV8-pLrat-sh*Nrp1* or control vector (sh-Ctrl). Hepatic fibrosis was induced by intraperitoneal (i.p.) injection of CCl_4_ (5 mL/kg of 10% CCl_4_ dissolved in olive oil, 3 times/week) for 4 weeks. Control groups (Vehicle) received an equal volume of olive oil via i.p. injection. A subset of *Nrp1*-knockdown mice was treated with the Rab7 inhibitor CID 1067700 (Rab7i, 10 mg/kg, i.p., 2 times/week) starting from week 1. **B** Representative images of H&E staining, Sirius Red staining, and immunohistochemistry for α-SMA and TβRI in serial liver sections from the indicated groups. **C** Quantification of Sirius Red-positive areas. **D** Serum levels of alanine aminotransferase (ALT) and aspartate aminotransferase (AST). **E** Western blot analysis of α-SMA and Collagen I expression in liver tissues. **F** Western blot analysis of NRP1, α-SMA, Collagen I, TβRI, p-Smad2/3, total Smad2/3, and Rab7 in freshly isolated primary HSCs from the indicated experimental groups. **G** Representative immunofluorescence images showing the expression of TβRI (green) and α-SMA (red) in primary HSCs freshly isolated from the indicated experimental groups. Data are presented as the mean ± SD. Statistical significance was determined by one-way ANOVA followed by Tukey’s multiple comparisons test. ns, not significant; * *p* < 0.05; ** *p* < 0.01; *** *p* < 0.001.

To evaluate the regulatory role of NRP1 in liver fibrosis in vivo, we achieved HSC-specific knockdown of *Nrp1* using AAV8-pLrat-sh*Nrp1* and performed functional rescue experiments with the Rab7 inhibitor CID 1067700 (Rab7i) (Fig. 6A). Histological analysis revealed that *Nrp1* knockdown significantly attenuated CCl_4_-induced hepatic collagen deposition; however, co-administration of Rab7i partially abrogated the protective effects conferred by NRP1 deficiency, leading to increased collagen accumulation (Fig. 6B, C). IHC analysis of serial liver sections further demonstrated that TβRI expression was markedly downregulated within α-SMA-positive fibrous septa in the *Nrp1* knockdown group, whereas Rab7i treatment partially restored TβRI-positive signals (Fig. 6B). In addition, serum ALT and AST levels further supported these phenotypic changes (Fig. 6D).

To further assess fibrosis severity in liver tissues, we performed Western blot analysis. The results showed that *Nrp1* knockdown significantly reduced the protein levels of α-SMA and Collagen I, whereas this effect was partially reversed by Rab7i treatment (Fig. 6E). In primary HSCs freshly isolated from these experimental mice, Western blotting confirmed that *Nrp1* knockdown was accompanied by decreased TβRI protein levels and reduced downstream p-Smad2/3 signaling. In contrast, Rab7i treatment maintained TβRI expression, thereby restoring TGF-β signaling and the activated HSC phenotype (Fig. 6F). Furthermore, immunofluorescence analysis in primary HSCs revealed that intracellular TβRI signals were markedly diminished upon *Nrp1* knockdown, and this reduction was partially rescued by Rab7i treatment (Fig. 6G). Collectively, these in vivo findings indicate that NRP1 deficiency promotes Rab7-dependent TβRI degradation, thereby alleviating the progression of liver fibrosis.

## Discussion

**Fig. 7:**
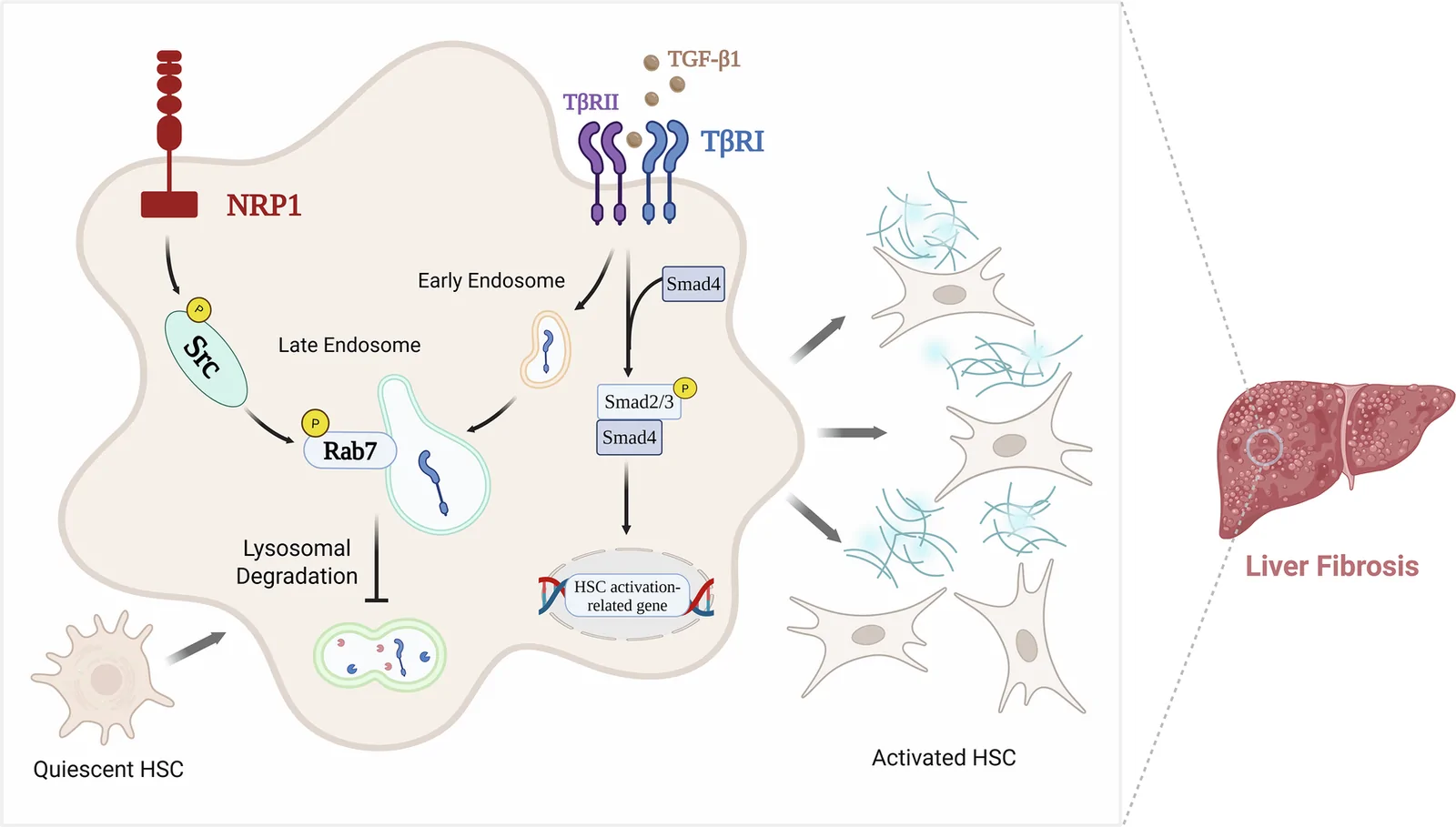
Schematic illustration of the mechanism by which NRP1 promotes liver fibrosis via the Src/Rab7 axis. In HSCs, NRP1 promotes the phosphorylation of Src and its downstream effector Rab7. This signaling cascade inhibits the lysosomal degradation of TβRI by modulating its endosomal trafficking, thereby sustaining TGF-β1/Smad2/3 signaling. The resulting transcriptional activation of fibrogenic genes drives the transition of quiescent HSCs into activated myofibroblasts, leading to the progression of liver fibrosis.

Liver fibrosis is a pivotal stage in the progression of chronic liver disease and can ultimately lead to cirrhosis, liver failure, and hepatocellular carcinoma. However, effective anti-fibrotic therapies remain limited, largely due to the complex and multifactorial nature of fibrogenesis. Building on our previous observation that NRP1 is upregulated in fibrotic livers and that NRP1 depletion attenuates HSC activation and fibrosis, the present study defines a mechanistic link between NRP1 and TGF-β signaling. We show that NRP1 promotes Src activation to promote Rab7 tyrosine phosphorylation, thereby limiting Rab7 activation and reducing Rab7-dependent lysosomal delivery of TβRI. This stabilizes TβRI, sustains Smad signaling, and reinforces the activated HSC phenotype (Fig. 7). Importantly, HSC-targeted *Nrp1* silencing in vivo promotes TβRI lysosomal degradation and markedly alleviates CCl_4_-induced liver fibrosis, highlighting the NRP1/Src/Rab7 axis as a potential therapeutic vulnerability.

NRP1 is a multifunctional single-pass transmembrane glycoprotein that was originally identified as a co-receptor for axon guidance cues such as semaphorins [18]. Subsequent studies have established NRP1 as an integrator of multiple signaling axes, with important roles in angiogenesis, immune regulation, and tissue fibrosis [7, 8]. Recent injury models further underscore its pathogenic role in fibrosis across organs. In renal ischemia-reperfusion injury, NRP1 is markedly upregulated in injured distal tubular cells and promotes the interaction between TNF-α and its receptor, thereby exacerbating renal injury and driving tubulointerstitial fibrosis [19]. Following myocardial infarction, NRP1 contributes to fibroblast activation and tissue remodeling; although it may support angiogenesis at early stages, sustained NRP1 signaling leads to excessive collagen deposition and aggravates ventricular wall fibrosis [20]. Consistent with these cross-organ observations, our data support a pro-fibrotic role for NRP1 in the liver: NRP1 enhances TGF-β1/Smad signaling and promotes HSC activation. Notably, *Nrp1* silencing reduced p-Smad2/3 levels and decreased TβRI protein levels without altering TβRI mRNA, whereas TβRII remained unchanged at both the mRNA and protein levels. Previously, NRP1 was thought to facilitate receptor activation primarily by binding ligands such as TGF-β1, VEGFA, and PDGF at the cell surface [21–25]. However, current evidence suggests that NRP1 may also regulate the intracellular transport and degradation of TβRI protein. By controlling the cellular abundance of this receptor, NRP1 thereby determines the intensity and duration of downstream signaling.

In the TGF-β signaling pathway, the sorting of receptors after they enter the cell determines the strength and duration of the signal [13]. After internalization, receptors move into Rab5-positive early endosomes to maintain Smad signaling [15, 26]. From there, they follow one of two routes: they are either sent to Rab7-positive late endosomes for lysosomal degradation or routed to Rab11-positive recycling endosomes to return to the cell membrane [26, 27]. Therefore, the processes of internalization, sorting, and recycling serve as essential regulatory steps for TGF-β signaling. Rab small GTPases coordinate vesicle movement and tethering as molecular switches. Rab7 is a major regulator of late endosome-to-lysosome transport and can bias receptors toward the degradative pathway [26, 27]. Active Rab7 may regulate this degradative route through downstream effector proteins that coordinate vesicle motility, organelle positioning, and late endosome–lysosome fusion [28]. In this context, our data support the view that NRP1 acts upstream to influence the distribution of TGF-β receptor trafficking fates. Loss of NRP1 increases the association of TβRI with Rab7 and is accompanied by enhanced lysosomal delivery. These changes coincide with reduced TβRI protein levels and lower TGF-β1/Smad signaling output. Conversely, NRP1 is associated with decreased Rab7-dependent lysosomal targeting, which may help maintain TβRI homeostasis and support sustained activation of HSCs. The specific adaptor molecules or trafficking regulators linking TβRI-containing vesicles to Rab7-positive compartments have yet to be identified. Prior work indicates that Rab7 activity can be modulated by phosphorylation [29] and that Src-mediated tyrosine phosphorylation of Rab7 alters its function in vesicular trafficking [30]. Previous studies have suggested that NRP1 can participate in ABL/Src family kinase signaling [31]. In our study, NRP1 loss reduced Src phosphorylation and Src-dependent Rab7 phosphorylation, accompanied by increased Rab7-GTP levels. These findings suggest that NRP1 may restrain Rab7-driven lysosomal sorting and degradation of TβRI through Src-dependent regulation of Rab7. Further studies are needed to clarify the precise mechanism by which NRP1 regulates Src activation. Taken together, our findings link an NRP1/Src/Rab7 axis to receptor trafficking control and suggest a mechanism by which NRP1 may help sustain TGF-β signaling during liver fibrogenesis.

Our findings have translational relevance. Since TGF-β receptors are widely expressed and contribute to essential physiological functions, systemic inhibition of these receptors as an anti-fibrotic strategy raises safety concerns and increases the risk of off-target effects [32]. This highlights the value of cell-targeted, mechanism-based interventions. AAV vectors have been increasingly used as gene-delivery platforms in clinical studies, in part because they show low tumorigenic potential and relatively limited immunogenicity [33, 34]. Based on this rationale, we used an AAV8 in vivo gene-manipulation strategy to silence *Nrp1* selectively in HSCs. Relative to control animals, mice receiving AAV8-mediated *Nrp1* silencing exhibited significant attenuation of liver fibrosis, with reduced collagen deposition and improved histopathological features. Together, these in vivo results support a pathogenic contribution of NRP1 to fibrogenesis and suggest that targeting NRP1 in HSCs may offer a feasible strategy to modulate receptor trafficking and reduce sustained profibrotic TGF-β signaling.

Several limitations of this study warrant further consideration. First, our in vivo evidence is primarily based on the CCl_4_-induced fibrosis model. Future studies should investigate whether the NRP1/Src/Rab7 axis plays a similar role in other etiologies, such as NASH or cholestasis. Second, while our data suggest a mechanism intrinsic to HSCs, NRP1 is also expressed in other hepatic cell types. The potential impact of microenvironmental factors, including immune cell recruitment and vascular remodeling, remains to be clarified using more stringent HSC-specific genetic models. Third, although we identified Src-dependent regulation of Rab7 as an important factor in TβRI lysosomal routing, several biochemical details require further validation. These include the relevant Rab7 phosphorylation sites, downstream effectors, and the timing and subcellular localization of these events along the endosomal pathway. Finally, the systemic administration of Rab7 inhibitors may cause off-target effects, as Rab7 is essential for basal lysosomal function and autophagy. Future clinical translation will require more cell-specific delivery strategies to minimize potential systemic toxicity.

In summary, our data indicate that NRP1 contributes to the maintenance of TGF-β/Smad signaling in HSCs. We propose a model in which NRP1 promotes Src activity and Src-dependent phosphorylation of Rab7. This is associated with reduced Rab7-GTP levels and decreased Rab7-mediated delivery of TβRI to lysosomes, which helps limit receptor degradation and maintain TβRI levels. By influencing this trafficking step, the NRP1/Src/Rab7 pathway may affect how long TGF-β signaling is sustained and, in turn, support ongoing HSC activation during chronic liver injury. Consistent with this interpretation, AAV-based silencing of *Nrp1* in HSCs reduced fibrosis severity in vivo, suggesting that modulating NRP1-regulated receptor trafficking could be explored as a therapeutic approach.

## Materials and methods

### Animals

Male C57BL/6 mice (6–8 weeks old) were obtained from the Laboratory Animal Center of Chongqing Medical University and housed under specific pathogen-free conditions. To induce hepatic fibrosis, mice received intraperitoneal (i.p.) injections of 10% CCl_4_ (5 mL/kg) three times weekly for four weeks, while control mice received an equal volume of olive oil. For HSC-specific gene silencing, a single tail vein injection of adeno-associated virus (AAV)-pLrat-sh*Nrp1* (GenePharma, Shanghai) or an empty vector was administered at day 0. The AAV8 vector carried an shRNA targeting *Nrp1* (5’-CCTGCTTTCTTCTCTTGGTTT-3’) driven by the Lrat promoter. To evaluate the rescue effect, the Rab7 inhibitor CID 1067700 (i.p., 10 mg/kg, twice weekly) was administered starting from the second week of CCl_4_ treatment for a total of three weeks. On Day 28, liver tissues were harvested for histological and molecular analyses. All experimental protocols were approved by the Ethics and Animal Care Committees of Chongqing Medical University (IACUC-CQMU-2025-01060).

### Primary HSC isolation and transfection

Primary mouse HSCs were isolated using in situ liver perfusion followed by enzymatic digestion with collagenase IV (0.5 mg/mL; Gibco). The resulting cell suspension was purified via OptiPrep™ density-gradient centrifugation as previously described [35, 36]. Isolated HSCs were cultured in DMEM containing 3% FBS at 37 °C in a 5% CO_2_ atmosphere. The purity of isolated HSCs was confirmed by the presence of glial fibrillary acidic protein (GFAP) and by α-SMA expression after activation.

For signaling analysis, cells were stimulated with recombinant TGF-β1 (5 ng/mL; MCE). Transient transfections with shRNA plasmids (sh-Ctrl, sh-*Nrp1*) or siRNAs (si-Ctrl, si-*Rab7*) were performed using Lipofectamine 3000 (Thermo Fisher Scientific) according to the manufacturer’s protocol. The sense sequence for si-Rab7 was 5’-CAGTAACCAGTACAAAGCCACAATA-3’. A non-targeting sequence (sh-Ctrl or si-Ctrl) was used as a negative control.

### Antibodies and reagents

Antibodies used in this study were as follows: anti-α-SMA (GTX629702) from GeneTex; anti-Collagen I (91144), anti-phospho-Smad2/3 (8828), anti-Smad2/3 (8685), anti-phospho-Tyrosine (96215), anti-phospho-Src (Tyr416) (2101), and anti-Src (2108) from Cell Signaling Technology; anti-TβRII (ab269279), anti-LAMP1 (ab208943), and anti-NRP1 (ab81321) from Abcam; anti-TβRI (sc-518086), and anti-Rab7 (sc-376362) from Santa Cruz; anti-GAPDH (ET1601-4) from HUABIO; anti-rabbit IgG (511203) and anti-mouse IgG (511103) from Zenbio.

The following reagents and compounds were used in this study: CID 1067700 (SML0545, Sigma-Aldrich), Src-activating peptide (EPQpYEEIPIYL, HY-P3279, MCE), cycloheximide (HY-12320, MCE), chloroquine (HY-17589A, MCE), and MG132 (HY-13259, MCE).

### qRT-PCR and Western blot

Total cellular RNA was isolated using TRIzol reagent according to the manufacturer’s instructions. The relative mRNA abundance of *Nrp1*, *Tgfbr1*, and *Tgfbr2* was quantified using the 2^–ΔΔCt^ method, with normalization to internal control genes. The oligonucleotide primers used in this study are listed in Table 1.

**Table 1:** Characterization of the sequences of cDNA primers.

Gene	Forward (5′-3′)	Reverse (5′-3′)
*Nrp1*	CCGATTCAGGACCATACAGG	TAGACCACAGGGCTCACCAG
*Tgfbr1*	TGGGACTTGCTGTGAGACAT	ATGTCAGCGCGTTTGAAGGA
*Tgfbr2*	AACGACTTGACCTGTTGCCT	TCCGTCTGCTTGAACGACTC
*GAPDH*	AACACGGAAGGCCATGCCA	GCATCCTGCACCACCAACTT

Protein lysates were prepared from tissues or cells using a specialized lysis buffer containing protease and phosphatase inhibitors. After determining protein concentrations with a BCA kit, equal amounts of protein were loaded onto SDS-PAGE gels for separation. The separated proteins were then transferred to PVDF membranes. The membranes were blocked and sequentially incubated with the indicated primary and secondary antibodies. Protein bands were visualized with ECL reagents, and the intensity of each band was quantified using densitometry software. To normalize the data, GAPDH was utilized as an internal reference.

### Immunoprecipitation (IP) and pull-down assays

To examine endogenous protein associations, cell lysates were subjected to co-immunoprecipitation (Co-IP) by overnight incubation with primary antibodies targeting TβRI or Rab7 at 4°C. The resulting complexes were captured using Protein A/G magnetic beads and subsequently analyzed via immunoblotting. The activation status of Rab7 was assessed using a Rab7 Activation Assay Kit (NewEast Biosciences, 82501), which employs a conformation-specific monoclonal antibody to selectively immunoprecipitate the active, GTP-bound form of Rab7 from cell lysates. The captured Rab7-GTP complexes and total Rab7 from the input lysates were subsequently quantified by Western blot analysis to evaluate relative Rab7 activity. For the detection of Rab7 tyrosine phosphorylation, total Rab7 was immunoprecipitated and then probed with a specific anti-phospho-tyrosine (p-Tyr) antibody.

### Immunofluorescence (IF) and colocalization analysis

For immunocytochemical analysis, HSCs were fixed in 4% PFA and permeabilized with 0.1% Triton X-100. After blocking nonspecific binding, cells were sequentially incubated with primary and fluorophore-conjugated secondary antibodies. DAPI was employed to label the nuclei. All microscopic images were acquired with consistent exposure and gain settings to ensure intergroup comparability. Quantitative colocalization analysis, expressed as Pearson’s correlation coefficient (R), was performed using ImageJ.

### Histological analysis

Liver specimens were fixed in 4% paraformaldehyde, followed by dehydration and embedding in paraffin. Thin sections (5 µm) were cut for routine Hematoxylin and Eosin (H&E) staining to examine hepatic morphology. To quantify the fibrotic area and collagen accumulation, Sirius Red staining was performed. For immunohistochemical (IHC) analysis, paraffin-embedded slides underwent deparaffinization and rehydration, followed by heat-induced antigen retrieval and blocking of nonspecific binding. The sections were subsequently incubated with designated primary antibodies and visualized through standard peroxidase-based chromogenic development.

### Statistical analysis

Data are expressed as mean ± SD from at least three independent experiments. Comparisons between two groups were performed using a two-tailed Student’s *t* test. Multiple group comparisons were analyzed by one-way ANOVA followed by Tukey’s post-hoc test. Protein degradation curves were analyzed by two-way repeated measures ANOVA followed by Sidak’s multiple comparisons test. Statistical significance was defined as *p < 0.05, **p < 0.01, and ***p < 0.001.

## Supplementary information

Western blot
