# Supplementary material for: NRP1 promotes hepatic fibrosis by regulating Src/Rab7-mediated lysosomal degradation of TβRI
Source: Cell Death Discov. 2026 Jun 28;12:370. doi: 10.1038/s41420-026-03220-w (PMC13572504; doi:10.1038/s41420-026-03220-w)
Supplement: Supplementary file 1 [file 41420_2026_3220_MOESM1_ESM.docx]

Fig 1

Fig 2

Fig 3

Fig 4

Fig 5

Fig 6
